# Azaspiracids Increase Mitochondrial Dehydrogenases Activity in Hepatocytes: Involvement of Potassium and Chloride Ions

**DOI:** 10.3390/md17050276

**Published:** 2019-05-08

**Authors:** Marco Pelin, Jane Kilcoyne, Chiara Florio, Philipp Hess, Aurelia Tubaro, Silvio Sosa

**Affiliations:** 1 Department of Life Sciences, University of Trieste, Via A. Valerio 6, 34127 Trieste, Italy; mpelin@units.it (M.P.); florioc@units.it (C.F.); tubaro@units.it (A.T.); 2 Marine Institute, Rinville, Oranmore, Co. H91 R673 Galway, Ireland; jane.kilcoyne@marine.ie; 3 IFREMER, Laboratoire Phycotoxines, Rue de l’Ile d’Yeu, 44311 Nantes, France; philipp.hess@ifremer.fr

**Keywords:** azaspiracids, hepatocytes, mitochondrial activity, mechanism of toxicity

## Abstract

Background: Azaspiracids (AZAs) are marine toxins that are produced by *Azadinium* and *Amphidoma* dinoflagellates that can contaminate edible shellfish inducing a foodborne poisoning in humans, which is characterized by gastrointestinal symptoms. Among these, AZA1, -2, and -3 are regulated in the European Union, being the most important in terms of occurrence and toxicity. In vivo studies in mice showed that, in addition to gastrointestinal effects, AZA1 induces liver alterations that are visible as a swollen organ, with the presence of hepatocellular fat droplets and vacuoles. Hence, an in vitro study was carried out to investigate the effects of AZA1, -2, and -3 on liver cells, using human non-tumor IHH hepatocytes. Results: The exposure of IHH cells to AZA1, -2, or -3 (5 × 10^−12^–1 × 10^−7^ M) for 24 h did not affect the cell viability and proliferation (Sulforhodamine B assay and ^3^H-Thymidine incorporation assay), but they induced a significant concentration-dependent increase of mitochondrial dehydrogenases activity (MTT reduction assay). This effect depends on the activity of mitochondrial electron transport chain complex I and II, being counteracted by rotenone and tenoyl trifluoroacetone, respectively. Furthermore, AZAs-increased mitochondrial dehydrogenase activity was almost totally suppressed in the K^+^-, Cl^−^-, and Na^+^-free media and sensitive to the specific inhibitors of K_ATP_ and hERG potassium channels, Na^+^/K^+^, ATPase, and cystic fibrosis transmembrane conductance regulator (CFTR) chloride channels. Conclusions: These results suggest that AZA mitochondrial effects in hepatocytes derive from an imbalance of intracellular levels of K^+^ and, in particular, Cl^−^ ions, as demonstrated by the selective reduction of toxin effects by CFTR chloride channel inhibition.

## 1. Introduction

Azaspiracids (AZAs) are polyether marine toxins that are produced by dinoflagellates of the genera *Azadinium* and *Amphidoma.* These toxins can accumulate in filter feeding edible shellfish, whose ingestion can induce a seafood poisoning, named Azaspiracid Shellfish Poisoning (AZP) [1]. The first AZP case was described in 1995, when, after the consumption of contaminated Irish mussels, eight people experienced gastrointestinal symptoms, including nausea, vomiting, and diarrhea, similar to those of Diarrhetic Shellfish Poisonings (DSP) [2]. A few years later, a novel toxin, initially called Killary-toxin and subsequently renamed AZA1, was identified in the contaminated shellfish [3]. Subsequently, following a second poisoning incident in 1997 in Arranmore Island (Ireland), the 8-methyl and 22-demethyl azaspiracid derivatives were identified and called AZA2 and AZA3, respectively [4]. Currently, more than 40 analogues have been identified, with the majority of them being produced by dinoflagellates or derived from shellfish metabolism [1]. However, only AZA1, -2, and -3 are regulated in the European Union, where the maximum allowed level in seafood has been established at 160 μg of AZA1 equivalents per kg of shellfish meat [5,6].

Human poisonings that were ascribed to AZAs are currently limited to the ingestion of contaminated mussels. Following the first episodes of Killary Harbour and Arranmore Island (Ireland), several cases have been described. In 1998, 10 people were poisoned after the consumption of Irish mussels in Italy and almost 30 people in France. Similar poisonings occurred in England (2000), Denmark (2002), and United States of America (USA) (2008), after the ingestion of Irish mussels [7]. The presence of AZAs and/or AZA-producing dinoflagellates has been documented in different geographical areas, such as Norway, France, Spain, Italy, Portugal, Argentina, and Mexico [8,9,10,11,12], making AZAs an emerging worldwide problem; however, to date, all AZA poisoning events have been associated with shellfish originating from Ireland.

Since the discovery of AZA1, several in vivo studies have been performed to characterize its toxicity, after both oral and intraperitoneal (i.p.) administration. In general, AZAs cause damage in different organs, as observed at the intestinal, gastric, hepatic, pancreatic, pulmonary, and splenic level [8]. In particular, i.p. injection in mice of a semi-purified AZA extract induced the swelling of the liver, which was characterized by a distinctive yellowish hue. In addition, histopathological analysis showed fat droplets in the hepatocytes cytoplasm and vacuoles in the centro-lobular and sub-capsular regions of the liver [13]. Similarly, an increased (38%) liver weight was observed in mice within 24 h after the acute oral administration of purified AZA1 (500 μg/kg), whereas the accumulation of fat droplets in the hepatocytes was observed after 1 h exposure to the dose of 300 μg/kg [14]. Liver damage was also demonstrated by other studies after single oral administration of AZA1 [15,16], as well as after repeated oral exposure to AZA1 in mice, which was shown to induce accumulation of fat droplets in hepatocytes, as well as focal and single-cell necrosis [17]. In addition, a toxicokinetic study in mice after the acute oral administration of sub-lethal doses of AZA1 (100–300 μg/kg), detected the highest amount of toxin in the liver, followed by kidneys, lungs, spleen, and heart, even though significant tissue damage was only observed at the intestinal level [18]. Recently, a comparative acute oral toxicity study in mice on AZA1, -2, and -3 confirmed the hepatotoxic effects of these toxins, as revealed by the enlarged pale liver, hepatocytes necrosis, and increased serum markers of hepatic alteration [19]. Therefore, when considering the liver as one of the target organs of AZAs, an in vitro study was carried out to investigate the effects of AZA1, -2, and -3 on human hepatocytes, using the IHH non-tumor cell line.

## 2. Results

### 2.1. Cytotoxicity of Azaspiracids towards IHH Cells

**Figure 1:**
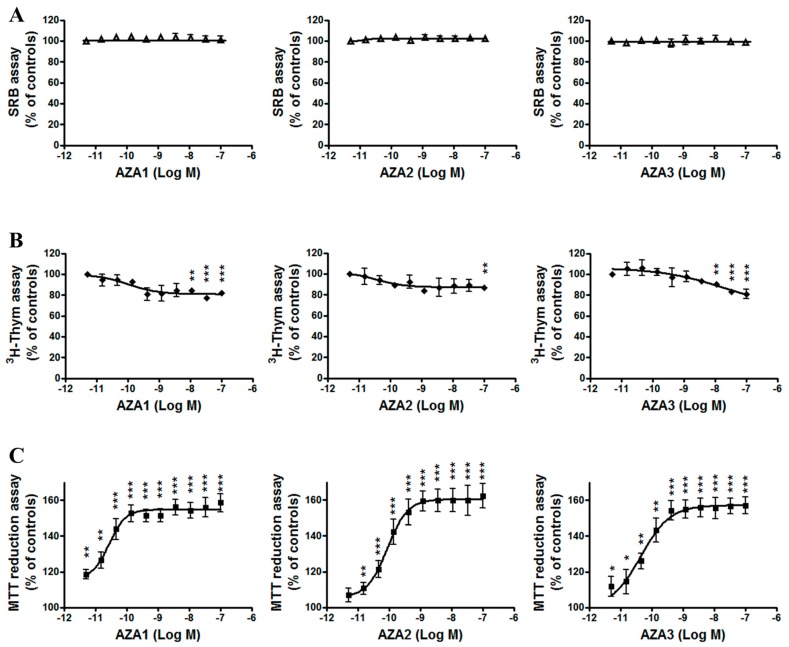
Effects of Azaspiracid-1 (AZA1), -2, and -3 on IHH cells viability and proliferation evaluated by the SRB assay (panel **A**), the ^3^H-Thymidine incorporation assay (panel **B**) and the MTT assay (panel **C**) after 24 h exposure. Data are the means ± SE of five independent experiments performed in triplicate and they are reported as % of untreated control. Statistical differences *vs* untreated controls: *, p < 0.05; **, p < 0.01; ***, p < 0.001 (One-way ANOVA and Bonferroni’s post test).

The effect of 24 h IHH cells exposure to AZA1, -2, and -3 (5 × 10^−12^–1 × 10^−7^ M) was evaluated by the Sulforhodamine B (SRB) assay, the ^3^H-thymidine incorporation assay, and the MTT reduction assay (Figure 1). As compared to untreated control cells, the SRB assay (Figure 1, panel A) showed that toxins exposure did not exert any significant cytotoxic effect in IHH cells. The ^3^H-thymidine incorporation assay (Figure 1, panel B) showed only a slight reduction of cell proliferation: with respect to untreated controls, it was only significant at the concentration of 1 × 10^−8^ M and above for AZA1 and -3 (−18 and −19%, respectively; p < 0.01), and at the highest concentration for AZA2 (1 × 10^−7^ M, −13%; p < 0.01). On the contrary, the MTT assay showed a concentration-dependent increase of MTT reduction in cells that were exposed to AZAs: when compared to the untreated control cells, it was significant at the lowest concentration for AZA1 and -3 (5 × 10^−12^ M; p < 0.05) and at the concentration of 1 × 10^−11^ M (p < 0.01) for AZA2. The increased MTT reduction reached a plateau at the concentrations of 1 × 10^−10^ M for AZA1 (52% increase) and 1 × 10^−9^ M for AZA2 and -3 (53% and 54% increase, respectively).

On the whole, these results indicate that 24 h exposure of IHH hepatocytes to AZAs does not influence the cell viability or proliferation and that the increased MTT reduction can be ascribed to an increased activity of mitochondrial dehydrogenases, rather than to increased cell numbers.

### 2.2. Azaspiracids-Increased Mitochondrial Dehydrogenases Activity Depends on Mitochondrial Electron Transport Chain Complex I and II Function

**Figure 2:**
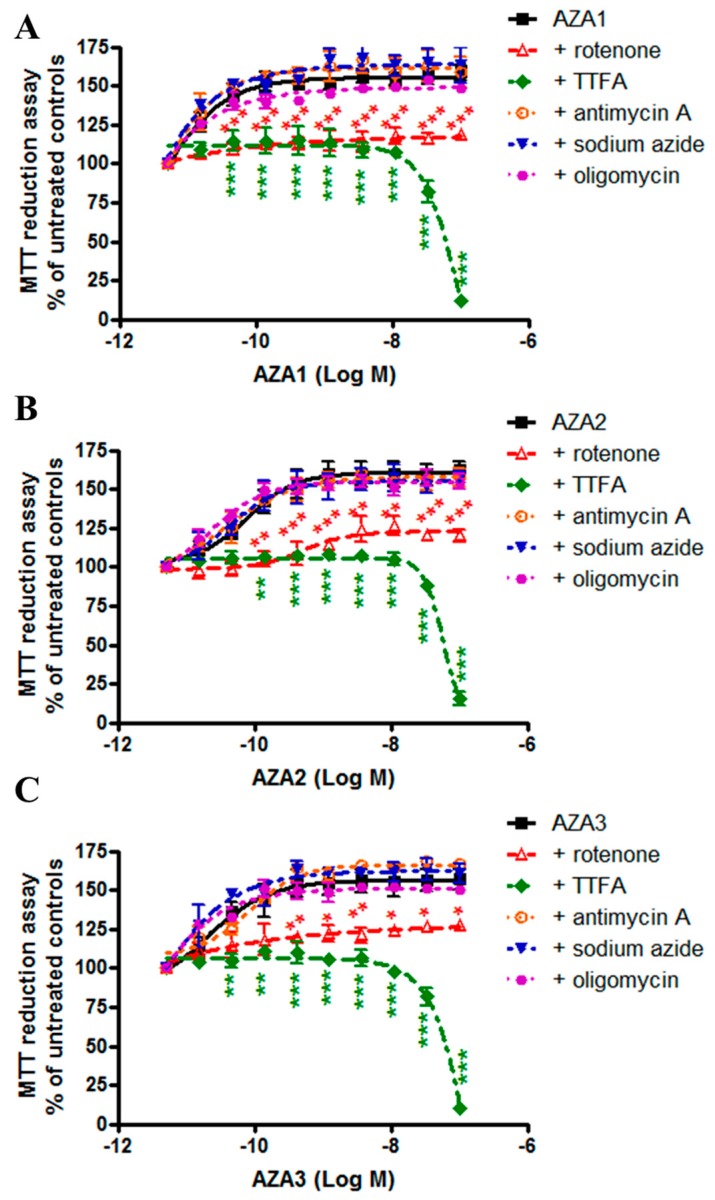
Role of mitochondrial electron transport chain on AZAs effect. IHH cells were exposed for 1 h to selective inhibitors of mitochondrial complexes, followed by 24 h exposure to AZA1 (A), -2 (**B**) or -3 (**C**), and the effects evaluated by the MTT assay. The results are expressed as % with respect to the untreated controls (for AZAs alone) and to untreated controls that were exposed to the specific inhibitor (for AZAs in association with the inhibitors). Data are the means ± SE of five independent experiments performed in triplicate and they are reported as % of untreated control. Statistical differences: *, p < 0.05; **, p < 0.01; ***, p < 0.001 (two-way ANOVA and Bonferroni’s post test).

To investigate whether the increased activity of mitochondrial dehydrogenases induced by AZAs was due to an increased function of mitochondrial electron transport chain, the IHH cells were exposed to the toxins in association with the selective inhibitors of mitochondrial chain complexes. In particular, the IHH cells were pre-exposed for 1 h to 5 × 10^−6^ M rotenone (inhibitor of complex I), 1 × 10^−3^ M tenoyl trifluoroacetone (TTFA, inhibitor of complex II), 2 × 10^−5^ M antimycin-A (inhibitor of complex III), 1.5 × 10^−4^ M sodium azide (inhibitor of complex IV), or 1 × 10^−6^ M oligomycin (inhibitor of ATP synthase), followed by 24 h co-exposure with AZAs (5 × 10^−12^–1 × 10^−7^ M), before the MTT assay. Figure 2 shows that the increased mitochondrial dehydrogenases activity induced by AZA1 (panel A), AZA2 (panel B), and AZA3 (panel C) was almost totally abolished by rotenone and by TTFA. In contrast to rotenone, the association of AZAs with TTFA resulted in a high reduction of mitochondrial activity, starting from the AZA concentration of 1 × 10^−8^ M, with a maximal reduction of 88%, 85%, and 90% for AZA1, -2, and -3, respectively. This effect could be ascribed to the cytotoxic potential of TTFA [20], which might be increased by high AZAs concentrations. All of the other inhibitors tested were ineffective.

These results suggest that the AZAs-increased mitochondrial dehydrogenase activity may be dependent on the activation of mitochondrial complexes I and II.

### 2.3. Azaspiracids-Increased Mitochondrial Dehydrogenases Activity Depends on H^+^ Gradient across the Mitochondrial Inner Membrane

The activity of mitochondrial complexes I and II is strictly dependent on the correct H^+^ gradient across the H^+^-impermeable mitochondrial inner membrane. Thus, we investigated whether dissipation of the proton gradient could prevent the increase of mitochondrial dehydrogenase activity induced by AZAs by exposing IHH cells to the toxins in association with nigericin, an ionophore that is able to revert the H^+^ gradient.

**Figure 3:**
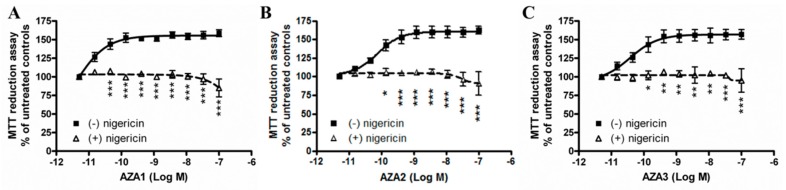
Role of H^+^ gradient on AZAs effect at the mitochondrial level. IHH cells were exposed for 1 h to 5 µM nigericin, followed by 24 h exposure to AZA1 (**A**), -2 (**B**), or -3 (**C**), and the effects evaluated by the MTT assay. Data are the means ± SE of five independent experiments that were performed in triplicate and are reported as % of untreated control. Statistical differences: *, p < 0.05; **, p < 0.01; ***, p < 0.001 (two-way ANOVA and Bonferroni’s post test).

Cells that were pre-exposed to 5 µM nigericin for 1 h were subsequently exposed to each AZA (5 × 10^−12^–1 × 10^−7^ M) for 24 h and submitted to the MTT reduction assay. As shown in Figure 3, nigericin eliminated the increased the mitochondrial dehydrogenase activity that was induced by AZA1, -2, and -3. This result suggests that AZA-increased mitochondrial dehydrogenases activity in IHH cells is strictly related to the H^+^ gradient across the inner mitochondrial membrane.

### 2.4. Azaspiracids Mitochondrial Effects Depend on Ionic Imbalance

**Figure 4:**
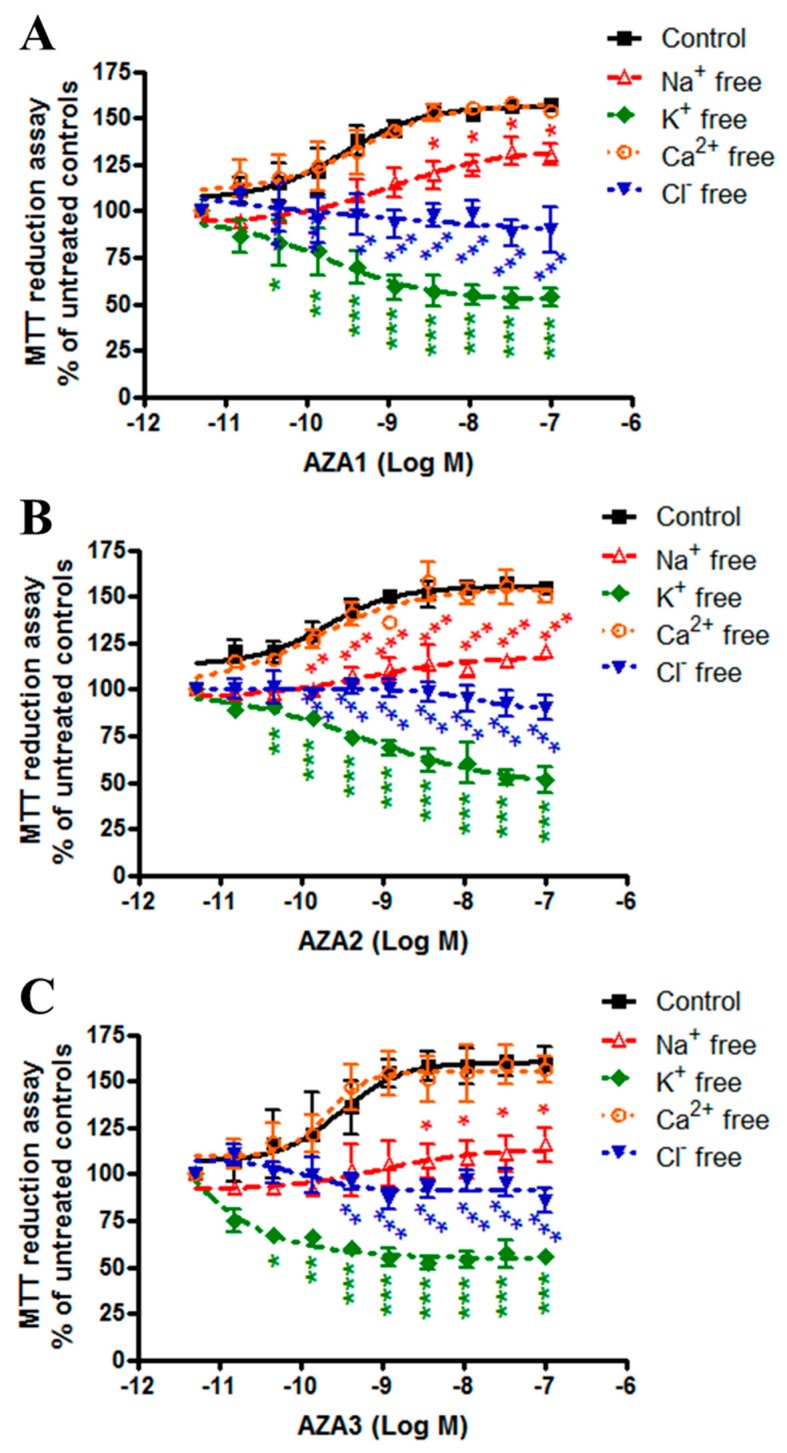
Role of ionic imbalance on AZAs effect at the mitochondrial level. IHH cells were exposed for 24 h to AZA1 (**A**), -2 (**B**), or -3 (**C**) in modified specific ion-free media, and the effects evaluated by the MTT assay. Data are the means ± SE of five independent experiments that were performed in triplicate and are reported as % of untreated control. Statistical differences: *, p < 0.05; **, p < 0.01; ***, p < 0.001 (two-way ANOVA and Bonferroni’s post test).

Since the H^+^ gradient across the mitochondrial inner membrane is maintained and tightly controlled by different ion transporters, we investigated the effect of selected ions withdrawal on AZAs-increased dehydrogenases activity. To this aim, the experiments were carried out exposing the IHH cells to the toxins in modified culture media, in which Na^+^, K^+^, Ca^2+^, or Cl^−^ ions were selectively withdrawn. For each ion-free medium, the % of MTT reduction in cells that were exposed to each AZA was calculated with respect to the untreated controls (cells not exposed to the toxins, cultured in each corresponding ion-free medium). The results were compared to the % of MTT reduction in cells that were exposed to each AZA in the medium containing all of the ions, calculated with respect to the corresponding untreated controls (cells not exposed to the toxins, cultured in the medium containing all of the ions) (Figure 4). In general, the increased mitochondrial dehydrogenases activity induced by AZAs was significantly reduced in K^+^-free, Cl^−^-free and, to a lesser extent, in Na^+^-free medium, but not in Ca^2+^-free medium. In particular, in the K^+^-free medium, this reduction was significant, starting from the concentration of 4 × 10^−11^ M for AZA1 (p < 0.05), -2 (p < 0.01) and -3 (p < 0.05). In addition, AZAs became cytotoxic in K^+^-free medium, reducing the mitochondrial dehydrogenase activity in a concentration-dependent way: at the highest concentration, AZA1, -2, and -3 induced a maximum reduction of 46, 48, and 44% with respect to the untreated controls, respectively. In the Cl^−^-free medium, the increased dehydrogenase activity was significantly reduced by AZAs starting from the concentration of 4 × 10^−10^ M (p < 0.01), 1 × 10^−10^ M (p < 0.001) and 4 × 10^−10^ M (p < 0.01) for AZA1, -2, and -3, respectively, whereas in the Na^+^-free medium, the reduction was significant, starting from the concentration of 4 × 10^−9^ M (p < 0.05), 1 × 10^−10^ M (p < 0.01), and 4 × 10^−9^ M (p < 0.05) for AZA1, -2, and -3, respectively. On the contrary, no inhibitory effects were observed in Ca^2+^-free medium.

These results suggest an involvement of monovalent ions (K^+^, Cl^−^, and Na^+^) in AZA-increased mitochondrial dehydrogenases activity, with the following rank of importance: K^+^ > Cl^−^ > Na^+^.

### 2.5. Role of K^+^ Transporters in Azaspiracids Effects

**Figure 5:**
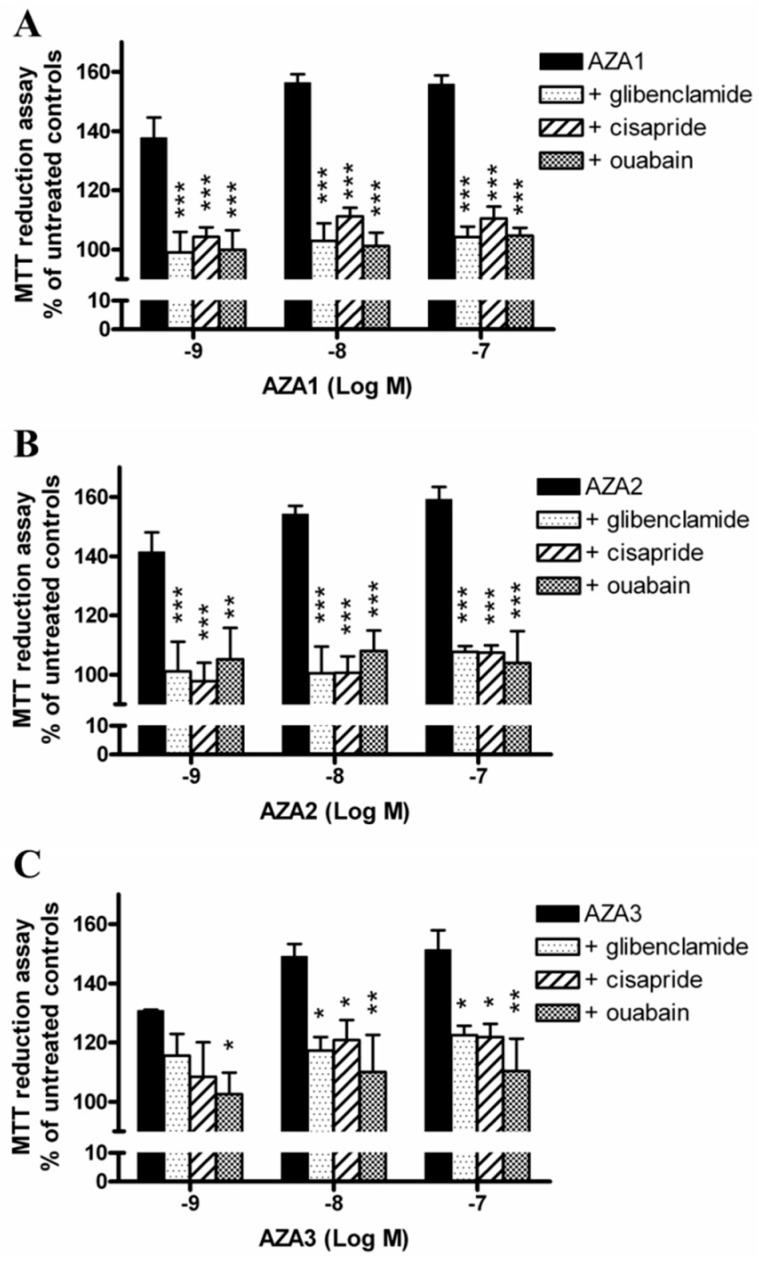
Role of K^+^ transporters on AZAs effect at the mitochondrial level. The IHH cells were exposed for 1 h to 100 µM glibenclamide (inhibitor of K_ATP_ channel), 5 µM cisapride (inhibitor of hERG channel) or 10 µM ouabain (inhibitor of Na^+^/K^+^-ATPase), followed by for 24 h exposure to AZA1 (**A**), -2 (**B**) or -3 (**C**), and the effects evaluated by the MTT assay. Results are expressed as % of mitochondrial activity with respect to the untreated controls (for AZAs alone) and to the untreated controls exposed to the specific inhibitor (for AZAs in co-association with the inhibitors). Data are the means ± SE of five independent experiments performed in triplicate and are reported as % of untreated control. Statistical differences: *, p < 0.05; **, p < 0.01; ***, p < 0.001 (two-way ANOVA and Bonferroni’s post test).

To further investigate the role of K^+^ on AZAs-increased mitochondrial dehydrogenase activity, the experiments were carried out exposing IHH cells to the toxins in the presence of selective inhibitors of K^+^ transporters that are expressed in hepatocytes. In particular, the cells were pre-exposed for 1 h to 100 µM glibenclamide (inhibitor of K_ATP_ channel), 5 µM cisapride (inhibitor of hERG channel), or 10 µM ouabain (inhibitor of Na^+^/K^+^-ATPase), followed by 24 h exposure to each AZA (1 × 10^−9^–1 × 10^−7^ M). Subsequently, mitochondrial dehydrogenases activity was evaluated by the MTT reduction assay (Figure 5). The results are expressed as % of mitochondrial dehydrogenase activity in comparison to that recorded in the corresponding control cells that were exposed to each inhibitor, but not to the toxins. In general, all of the inhibitors significantly reduced the effects of AZAs to a similar extent, starting from the concentration of 1 × 10^−9^ M for all of the toxins. In particular, the mitochondrial dehydrogenases activity increased by the highest AZAs concentration (1 × 10^−7^ M) was reduced by glibenclamide, cisapride, and ouabain at levels that were comparable to those of the control cells, not exposed to the toxins: 104, 110, and 105%, respectively, for AZA1 (p < 0.001); 108, 107, and 104%, respectively, for AZA2 (p < 0.001); and, 122, 122, and 110%, respectively, for AZA3 (p < 0.05).

### 2.6. Role of Cl^−^ Transporters in Azaspiracids Effects

**Figure 6:**
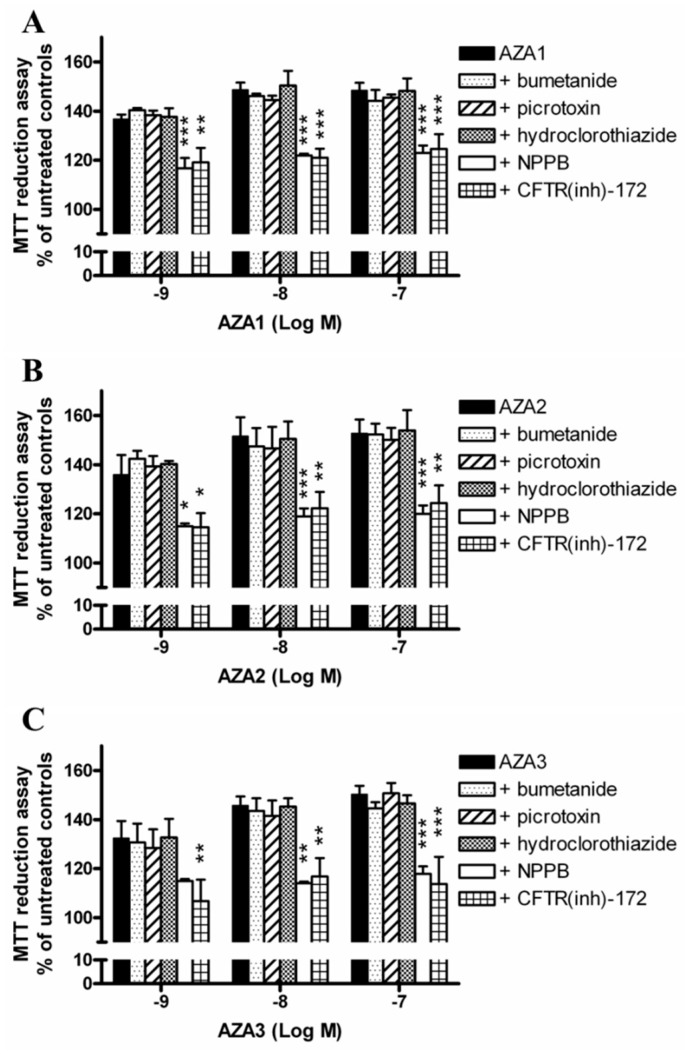
Role of Cl^−^ transporters on AZAs effect at the mitochondrial level. The IHH cells were exposed for 1 h to 100 µM NPPB (unspecific inhibitor of Cl^−^ channels), 5 µM CFTR(inh)-172 (inhibitor of CFTR), 100 µM picrotoxin (inhibitor of GABA_A_), 200 µM bumetanide (inhibitor of NKCC), or 100 µM hydrochlorothiazide (inhibitor of NCC), followed by for 24 h exposure to AZA1 (**A**), -2 (**B**), or -3 (**C**), and the effects evaluated by the MTT assay. The results are expressed as % of mitochondrial activity with respect to the untreated controls (with AZAs) and to untreated controls that were exposed to the specific inhibitor (with AZAs and inhibitors). Data are the means ± SE of five independent experiments performed in triplicate and they are reported as % of untreated control. Statistical differences: *, p < 0.05; **, p < 0.01; ***, p < 0.001 (two-way ANOVA and Bonferroni’s post test).

To investigate the role of Cl^−^ ions on AZAs-increased mitochondrial dehydrogenases activity, the experiments were carried out exposing IHH cells to each toxin and the inhibitors of selected Cl^−^ transporters. In particular, cells were pre-exposed for 1 h to 100 µM 5-nitro-2-(3-phenylpropyl-amino) benzoic acid (NPPB, unspecific inhibitor of Cl^−^ channels), 5 µM CFTR(inh)-172 (inhibitor of the cystic fibrosis transmembrane conductance regulator, CFTR), 100 µM picrotoxin (inhibitor of GABA_A_ receptors), 200 µM bumetanide (inhibitor of the Na^+^/K^+^/Cl^−^ co-transporter, NKCC), or 100 µM hydrochlorothiazide (inhibitor of Na^+^/Cl^−^ co-transporter, NCC), followed by 24 h exposure to each AZA (1 × 10^−9^–1 × 10^−7^ M). Subsequently, the mitochondrial dehydrogenase activity was evaluated by the MTT reduction assay (Figure 6). The results are expressed as % of mitochondrial dehydrogenase activity in comparison to that of the corresponding control cells that were exposed to each inhibitor, but not to the toxins. NPPB and CFTR(inh)-172 significantly reduced the increased mitochondrial dehydrogenases activity induced by AZAs, starting from the concentration of 1 × 10^−9^ M. In particular, the mitochondrial dehydrogenases activity increased by the highest concentration (1 × 10^−7^ M) of AZA1 (149%), AZA2 (153%), and AZA3 (150%) was significantly reduced at 123 and 124% (AZA1; p < 0.001), 120 and 124% (AZA2; p < 0.01), as well as 117 and 114% (AZA3; p < 0.001) by NPPB and CFTR(inh)-172, respectively. On the contrary, the other inhibitors were ineffective.

## 3. Discussion

The main symptoms of AZP are at gastrointestinal level (nausea, vomiting, diarrhea, and abdominal cramps), but in vivo studies on AZAs in mice also showed significant damage at the hepatic level, with the accumulation of fat droplets, vacuole formation, and liver swelling [13,14,17,18,19].

To investigate the effects of the regulated AZAs (AZA1, -2, and -3) on liver cells, an in vitro study was carried out using the non-tumor IHH human hepatocytes. Their effect was initially investigated by three methods, which were able to evaluate the mitochondrial dehydrogenases activity (MTT reduction assay), cytotoxicity (SRB assay), and cell proliferation (^3^H-thymidine incorporation assay). The MTT reduction assay, which was used to measure cell viability [21], showed a significant increase of the mitochondrial dehydrogenases activity in cells that were exposed for 24 h to the toxins, even at picomolar or sub-nanomolar concentrations. This effect, without signs of cytotoxicity, was very recently recorded only in undifferentiated intestinal Caco-2 cells that were exposed to nanomolar AZA1 concentrations [22]. The mechanism was not clarified and the authors speculated that it may be related to an altered coupling between glycolysis and the Krebs cycle. Herein, we confirmed the ability of AZAs to increase the MTT conversion in the absence of cytotoxic effects (SRB incorporation assay), also in liver cells. In addition, the increased MTT reduction seems to not be ascribable to a proliferative stimulus, since no increase of cell proliferation (^3^H-thymidine incorporation assay) was observed.

Thus, we verified whether the increased MTT reduction, as induced by AZAs, is due to a specific effect on mitochondrial electron transport chain function. Hence, the role of the four mitochondrial electron transport chain complexes was evaluated by MTT assay in IHH cells that were exposed to specific inhibitors of these complexes in association with AZAs. Among these inhibitors, only rotenone and TTFA reduced the mitochondrial dehydrogenases activity that was increased by AZAs, suggesting the involvement of complexes I and II of the mitochondrial electron transport chain in the toxin effect. These results are in line with a proteomic study reporting the ability of AZA1 to increase the expression of proteins that are involved in oxidative phosphorylation, including the NADH-dehydrogenase, namely complex I [23]. When considering that the functionality of mitochondrial complex I and II is tightly dependent on the H^+^ gradient across the mitochondrial inner membrane [24], the effects of AZAs were evaluated in the presence of nigericin, an ionophore that is able to dissipate the H^+^ gradient. Under this condition, the mitochondrial dehydrogenases activity increased by AZAs was totally reverted. This result suggests that 24 h exposure to AZAs can enhance mitochondrial NADH-dependent dehydrogenase activity in liver cells, in a manner that is strictly related to the H^+^ gradient.

The mitochondrial transmembrane H^+^ gradient is tightly controlled and balanced by a wide range of ions, in particular, K^+^, Na^+^, Ca^2+^, and, to a lesser extent, Cl^−^ ions [25,26]. An investigation into the effect of Na^+^, Ca^2+^, Cl^−^, and K^+^ ion withdrawal on AZAs-increased mitochondrial dehydrogenase activity found that, in IHH cells that were exposed to AZAs for 24 h in K^+^-, Na^+^-, and Cl^−^-free media, the toxins effects were significantly reduced, while Ca^2+^ withdrawal had no impact. These results suggest that AZA increased dehydrogenases activity is based on a mechanism that is strictly dependent on the presence of monovalent ions, in particular, K^+^ and Cl^−^ ions.

In addition, the removal of K^+^ ions not only reversed the toxin-dependent increase of mitochondrial dehydrogenases activity, but significantly decreased it to values that were below the control levels (44‒46% reduction with respect to untreated controls at the highest AZAs concentration), indicating the onset of a cytotoxic effect. We could not exclude that the withdrawal of ions could increase cell sensitivity to the cytotoxic activity of AZAs, when considering the highly stressful experimental condition, such as that induced by K^+^ removal from the medium. Furthermore, this condition is bound to force a redistribution of the ionic fluxes through the cells. Hence, we took a different approach, which examined the blockade of ions channels/transporters on AZAs effects using specific inhibitors. To this aim, we carried out an in-depth search on the Model Organism Protein Expression Database (MOPED) [27] to select the main K^+^ and Cl^−^ transporters that are expressed in hepatocytes. Among the K^+^ transporters, the ATP-sensitive inward rectifier K^+^ channel (K_ATP_ channels, also known as Kir6.x), the human ether-a-go-go-related gene channel (hERG, also known as Kv11.1), and the Na^+^/K^+^ ATPase were considered. As shown in Figure 5, all of the inhibitors significantly antagonized the effects of AZAs, further supporting a role for K^+^ ions in mediating the toxin effects. AZA2 has been previously shown to increase the hERG levels on the surface of hERG Chinese Hamster Ovary recombinant cells after 12 h exposure [28]. Moreover, a possible involvement of these channels in mediating AZAs cytotoxic effects has been suggested in a study showing that these toxins can act as hERG channels open-state blockers in the HEK-293 cells [29]. However, since the AZA concentrations needed to achieve channel blockade are in the micromolar range [29], it is unlikely that such a mechanism could be the main one that is responsible for the AZAs-mediated increase of dehydrogenases activity that was observed in the present study. On the contrary, and more reasonably, the elimination of AZAs effects on mitochondrial activity seems to be a consequence of an imbalance in the intracellular K^+^ ions derived from the blockade of K^+^ channels/pump, a condition that could resemble/mimic the ion withdrawal condition.

Finally, the role of Cl^−^ ions was investigated while using selective inhibitors of the cystic fibrosis transmembrane conductance regulator (CFTR), the GABA_A_ receptor-linked Cl^−^ channel, the Na^+^/Cl^−^ co-transporter (NCC) and the Na^+^/K^+^/Cl^−^ co-transporter (NKCC), and a non-selective inhibitor of several kinds of Cl^−^ channels, NPPB [30]. Among them, only the selective inhibitor of CFTR channels CFTR(inh)-172, and the non-selective compound NPPB caused a significant reduction of the AZAs-enhanced mitochondrial dehydrogenases activity. The CFTR channel, an integral membrane protein that belongs to the family of ABC transporters, has been reported to be involved in regulating mitochondrial functions, including the activity of mitochondrial complex I. Indeed, the inhibition of CFTR channels by both pharmacological blockade and RNA silencing has been found to reduce the dehydrogenase activity of complex I in T84 and Caco-2 cells [31]. When considering that all of the other inhibitors of Cl^−^ transporters/channels tested (i.e. bumetanide, hydrochlorothiazide and picrotoxin) were ineffective in reducing AZAs-increased mitochondrial dehydrogenases in IHH hepatocytes, the effect of CFTR inhibition on AZAs activity appears to be rather specific. CFTR has been reported to also act as a regulator of other Cl^−^ channels, such as Ca^2+^-activated Cl^−^ channels (CaCC) and volume regulated anion channels (VRAC) [30]. These channels are sensitive to NPPB, the non-selective inhibitor that significantly reduced AZAs activity in IHH cells. Thus, it seems conceivable that the increased mitochondrial dehydrogenase activity that is induced by AZAs in IHH hepatocytes is dependent on the maintenance of Cl^−^ ions fluxes, possibly and primarily through CFTR channels activity. Since these channels are also known to be involved in the trans-membrane fluid secretion [32], we cannot exclude their involvement in the liver enlargement and discoloration observed in mice that were orally exposed to AZAs [14,15,16,19]. However, the putative relationship between these Cl^−^-dependent AZAs-induced early events and the effects observed at the hepatic level in AZAs-treated mice remains to be determined.

## 4. Materials and Methods

### 4.1. Toxins

AZA1, -2, and -3 were extracted from *Mytilus edulis.* The purity of each toxin (>95 %) was confirmed by LC-MS/MS and NMR analyses, as previously reported [19]. The stock solutions were maintained at −20 °C, while the working solutions were freshly prepared in culture medium the day of each experiment.

### 4.2. Cell Culture

Dr. Gabriele Stocco obtained the immortalized human hepatic IHH cell line (University of Trieste). The cell line was characterized by a doubling time of approximately 24 h and it was maintained in Dulbecco’s modified Eagle’s medium (DMEM) high glucose with the addition of 10% fetal bovine serum (FBS), 1.25% l-glutamine (0.2 M), 1% penicillin (10,000 IU/mL), 10 mg/mL streptomycin, 1% Hepes buffer (1 M), 0.01% human insulin (10^−4^ M), and 0.04% dexamethasone (1 mg/mL). The cell cultures were maintained according to standard procedures in a humidified incubator at 37 °C and with 5% CO_2_, and the cell passages were performed once a week. For in vitro studies, the cells were seeded in 96-wells plates at a density of 5 × 10^3^ cells/well. All of the experiments were performed between cell passage 11 and 25.

All of the reagents for cell culture were purchased from Sigma-Aldrich (Milan, Italy), if not otherwise indicated.

### 4.3. MTT Reduction Assay

The IHH cells were exposed to AZA1, -2, or -3 (5 × 10^−11^–1 × 10^−7^ M) for 24 h and washed twice with PBS. Subsequently, the cells were exposed to the MTT reagent (final concentration: 0.5 mg/mL) in fresh medium to avoid any unspecific signal given by AZAs and MTT interaction. After 4 h, formazan crystals were solubilized by 100 μL DMSO and the absorbance was read by an Automated Microplate Reader EL 311s (Bio-Tek Instruments, Winooski, VT, USA) at 540/630 nm. Data are the means ± SE of five independent experiments that were performed in triplicate and they are reported as % of MTT reduction with respect to the untreated control cells (100% of cell viability).

### 4.4. Sulforhodamine B Incorporation Assay

The effects of AZAs on cell viability were evaluated by the sulforhodamine B (SRB) incorporation assay, as previously described [33,34]. Briefly, after 24 h exposure to AZA1, -2, or -3 (5 × 10^−11^–1 × 10^−7^ M), the cells were washed with PBS, fixed with 50% (*v/v*) trichloroacetic acid for 1 h at 4 °C and stained for 30 min with 0.4% SRB in 1% (*w/v*) acetic acid. After washing with 1% (*v/v*) acetic acid, the incorporated dye was dissolved by 200 µL of 10 mM TRIZMA base solution and the absorbance was read by an Automated Microplate Reader EL 311s (Bio-Tek Instruments, Winooski, VT, USA) at 570 nm. The data are the means ± SE of five independent experiments that were performed in triplicate and they are reported as % of cell viability after AZAs exposure with respect to the untreated control cells (100% cell viability).

### 4.5. [Methyl-^3^H] Thymidine Incorporation Assay

The effects of AZAs on cell proliferation were evaluated by the [Methyl-^3^H] thymidine incorporation assay, as previously described [35]. Briefly, the cells were exposed for 24 h to AZA1, -2, or -3 (5 × 10^−11^–1 × 10^−7^ M). After 19 h incubation, the cells were pulsed with [Methyl-^3^H] thymidine (2.5 μCi/mL) and the incubation was continued for additional 5 h. Cells were then collected and the radioactivity of the samples was determined by a liquid scintillation analyzer (Wallac 1450 Microbeta liquid scintillation counter; PerkinElmer, Milan, Italy). [Methyl-^3^H] thymidine incorporation, being quantified by the raw counts per minute (cpm), was expressed as the percent of maximal incorporation (untreated control cells, 100% cell proliferation) for each experimental condition (cpm treated cells ×100/cpm untreated control cells). Data are the means ± SE of three independent experiments that were performed in triplicate and they are reported as % of cell proliferation after AZAs exposure with respect to untreated control cells (100% cell proliferation).

### 4.6. Effect of AZAs on IHH Cells in Selected Ions-Free Culture Media

The role of extracellular Na^+^, K^+^, Ca^2+^, and Cl^−^ ions on AZA-effects at the mitochondrial level was evaluated by the MTT assay. After 48 h of stabilization in standard culture medium, the IHH cells were washed and cell medium was substituted with specific modified media, free of each selected ion, freshly prepared in-house (modified media composition is reported in Table 1). After 1 h equilibration, the cells were exposed to AZA1, -2, or -3 in modified specific ion-free media for 24 h, before the MTT reduction assay. The effects of AZAs in each ion-free medium were compared to those that were recorded in the standard medium containing all of the ions. For each medium (with or without ions), the results are expressed as % of MTT reduction with respect to the relevant control (cells not exposed to the toxins, cultured in medium with and without ions).

### 4.7. Statistical Analysis

The results are presented as mean ± SE from at least three independent experiments. Data were analysed by two-way ANOVA, followed by Bonferroni’s post test (Prism GraphPad, Inc.; San Diego, CA, USA), and significant differences were considered at p < 0.05.

## 5. Conclusions

On the whole, these results demonstrate that a relatively short time exposure (24 h) of IHH hepatocytes to AZAs increases the mitochondrial complex I and II dehydrogenase activity. This effect was found to be highly sensitive to the withdrawal of specific ions, in particular, on K^+^ and Cl^−^ ions. Whereas, K^+^ ions depletion seems to reduce the AZAs effect on mitochondrial activity and induce AZA cell toxicity, Cl^−^ ions appear to exert a significant role in mediating the effect of these toxins at the mitochondrial level in liver cells. In fact, an AZA-dependent increase in mitochondrial activity was selectively reduced by the blockade of CFTR chloride channels, which suggests a key role in modulating the effects of AZAs at the hepatic level. Since these channels are also known to be involved in the trans-membrane fluid secretion [32], their involvement in the liver enlargement and discoloration observed in mice that were orally exposed to AZAs [14,15,16,19] cannot be excluded.

## Figures and Tables

**Table 1:** Composition of modified ion-free culture media.

	Control (M)	Na^+^-free (M)	K^+^-free (M)	Ca^2+^-free (M)	Cl^−^-free (M)
**NaCl**	1.4 × 10^−1^	-	1.44 × 10^−1^	1.4 × 10^−1^	-
**KCl**	4.4 × 10^−3^	4.4 × 10^−3^	-	4.4 × 10^−3^	-
**CaCl_2_**	2.5 × 10^−3^	2.5 × 10^−3^	2.5 × 10^−3^	-	-
**MgSO_4_**	1.2 × 10^−3^	1.2 × 10^−3^	1.2 × 10^−3^	1.2 × 10^−3^	1.2 × 10^−3^
**KH_2_PO_4_**	1.2 × 10^−3^	1.2 × 10^−3^	-	1.2 × 10^−3^	1.2 × 10^−3^
**NaH_2_PO_4_**	-	-	1.2 × 10^−3^	-	-
**NaNO_3_**	-	-	-	-	1.4 × 10^−1^
**Ca(NO_3_)_2_**	-	-	-	-	2.5 × 10^−3^
***N*-methyl-d-Glucamine**	-	1.4 × 10^−1^	-	-	-
**HEPES**	10^−2^	10^−2^	10^−2^	10^−2^	10^−2^
**EGTA**	-	-	-	2 × 10^−3^	-
**Glucose**	4500 mg/L	4500 mg/L	4500 mg/L	4500 mg/L	4500 mg/L
**l-Glu**	1.25%	1.25%	1.25%	1.25%	1.25%
**Penicillin/Streptomycin**	1%	1%	1%	1%	1%
**Insulin**	10^−8^ M	10^−8^ M	10^−8^ M	10^−8^ M	10^−8^ M
**Dexamethasone (1 mg/mL)**	0.04%	0.04%	0.04%	0.04%	0.04%
